# Poster 2021: The incidence of adverse reactions induced by subcutaneous immunotherapy in an allergy center in Monterrey, México: a retrospective chart review

**DOI:** 10.1186/1939-4551-7-S1-P27

**Published:** 2014-02-03

**Authors:** Sandra Nora Gonzalez-Diaz, Alfredo Arias, Lorena Rangel, Rafael Perez-Vanzzini, Samuel Palma-Gomez

**Affiliations:** 1 Universidad Autonoma de Nuevo Leon, Hospital Universitario, Monterrey, Mexico; 2 Allergy and Clinical Immunology, Universidad Autonoma De Nuevo Leon - Hospital Universitario “Dr. Jose Eleuterio Gonzalez”, Monterrey, Mexico; 3 Universidad Autonoma de Nuevo León, Centro Regional De Alergía e Inmunología Clínica, Hospital Universitario “Dr. Jose Eleuterio Gonzalez”, Monterrey, Mexico

## Background

To investigate the incidence of local reactions and systemic reactions of subcutaneous immunotherapy in an Allergy Center in Monterrey, Nuevo León, México.

## Methods

We conducted a retrospective chart review of patients receiving subcutaneous immunotherapy in our allergy practice between November 2012 to June 2013 looking at the incidence of local and systemic allergic reactions.

## Results

A total of 8069 subcutaneous immunotherapy injections were applied between November 2012 to June 2013. Among them, 1071 local reactions (LR) were observed (13.2%). Of the total of LR, 1067 were considered small local reactions (Local reaction no larger than the size of the palm of the patient’s hand [<80 – 100mm]) (99.6%) and 4 were considered large local reactions (large local reaction larger than the size of the patient’s palm [> 80–100 mm]) (0.4%). 32 systemic reactions (0.4%) were observed. These SR included respiratory symptoms and cutaneous symptoms. We used the World Allergy Organization Subcutaneous Immunotherapy Systemic Reaction Grading System to classify the SR. 27 SR were classified as grade 1 (84.3%). 3 SR were classified as grade 2 (9.3%), characterized by respiratory symptoms (cough, wheezing) and responded to an inhaled bronchodilator. 2 SR were classified as grade 3 (6.4%) characterized by respiratory symptoms (cough, wheezing, shortness of breath) and did not respond to inhaled bronchodilator and required epinephrine treatment (0.02% of total injections). Neither grade IV nor V systemic reactions were observed.

## Conclusions

The prevalence of adverse reactions to subcutaneous immunotherapy observed in our study is similar to the reported worldwide. The most reactions are local, small and self limited. The low prevalence of systemic reactions and the absence of fatalities makes subcutaneous immunotherapy a safe therapy.

